# Electrical Pudendal Nerve Stimulation as an Initial Neuromodulation Strategy for Neurogenic Urinary Retention

**DOI:** 10.1016/j.euros.2026.07.008

**Published:** 2026-09-04

**Authors:** Tian Li, Jiayi Xu, Siyou Wang, Jianwei Lv, Zouqin Huang

**Affiliations:** a Shanghai Pudong Hospital of Traditional Chinese Medicine, Shanghai, China; b Shanghai Fourth Rehabilitation Hospital, Shanghai, China; c The Clinical Research Section, Shanghai Research Institute of Acupuncture and Meridian, Shanghai, China; d Shanghai Pudong New Area Gongli Hospital, Shanghai, China

**Keywords:** Neurogenic bladder, Urinary retention, Pudendal nerve, Neuromodulation, Sacral neuromodulation

## Abstract

**Background and objective:**

Chronic neurogenic urinary retention often requires assisted emptying and remains challenging to treat. We evaluated whether electrical pudendal nerve stimulation (EPNS) improves bladder emptying and compared short-term outcomes with a standard sacral neuromodulation (SNM) test stimulation strategy.

**Methods:**

In this prospective, multicenter, assessor-masked study, 70 adults with chronic refractory neurogenic nonobstructive urinary retention received either EPNS (12 sessions over 4 wk) or a standard 4-wk continuous SNM test phase using a percutaneously placed S3 lead. The primary outcome was the percentage change from baseline in postvoid residual urine volume (PVR) at 4 wk. Secondary outcomes included changes in International Consultation on Incontinence Questionnaire–Lower Urinary Tract Symptoms and Quality of Life scores. Analyses used linear mixed models; propensity score matching was prespecified as a sensitivity analysis.

**Key findings and limitations:**

At 4 wk, the adjusted PVR improvement rate was 68% with EPNS versus 51% with SNM (between-group difference 17 percentage points; 95% confidence interval 5–29; *p* = 0.008). Improvements in symptom and quality of life scores also favored EPNS (*p* < 0.001 for both). Limitations include nonrandom treatment allocation and short follow-up.

**Conclusions and clinical implications:**

Outpatient EPNS produced clinically meaningful short-term improvements in bladder emptying and patient-reported outcomes without implantation, supporting consideration of EPNS as an initial step in a stepped neuromodulation pathway for neurogenic urinary retention.

## Take Home Message

Outpatient electrical pudendal nerve stimulation produced greater short-term improvement than the sacral neuromodulation test phase in refractory neurogenic nonobstructive urinary retention. It may offer a less invasive preimplant step within a staged neuromodulation pathway.

ADVANCING PRACTICE **What does this study add?**

This study compares outpatient electrical pudendal nerve stimulation (EPNS) with the sacral neuromodulation test phase in refractory neurogenic nonobstructive urinary retention. The study adds clinically relevant comparative evidence supporting EPNS as a potential less invasive step before implantation.

**Clinical Relevance**

Electrical pudendal nerve stimulation could provide a minimally invasive outpatient option before sacral neuromodulation implantation for patients with refractory neurogenic urinary retention. Incorporating EPNS into a stepped neuromodulation pathway may allow clinicians to assess treatment response before proceeding to an implanted device. Longer-term randomized studies are needed to establish the durability of benefit and identify the patients most likely to respond. Associate Editor: Phe Véronique, MD.

**Patient Summary**

We compared outpatient pudendal nerve stimulation with a sacral nerve stimulation test in adults with neurogenic urinary retention. Pudendal stimulation improved bladder emptying more at 4 wk. Larger randomized studies should confirm these findings.

## 1 Introduction

Neurogenic nonobstructive urinary retention (NOUR) is a cross-cutting consequence of diverse neurologic disease or injury, characterized by chronic incomplete bladder emptying without anatomic outlet obstruction [1]. Elevated postvoid residual (PVR) volumes are linked to recurrent urinary tract infections, urolithiasis, upper urinary tract dilation, and progressive renal impairment, substantially impairing health and quality of life. Restoration of volitional voiding remains challenging, and many patients rely on catheter-assisted emptying [2]. Beyond individual burden, NOUR creates recurring care needs and ongoing device use across health care settings.

Sacral neuromodulation (SNM) is guideline-recommended for refractory NOUR [3], with meta-analyses reporting an 84% pooled success among permanent implants with meaningful PVR reductions [4], [5], [6]. Nevertheless, SNM requires surgical implantation with high upfront costs and is hampered by high long-term reintervention rates (eg, 69% reoperation over 23 yr; 42% device revision rate and a 17% explantation rate at 5.7 yr) [7], [8], [9], [10]. Moreover, response during the external test phase may not reliably predict 24-mo subjective success (c-index <0.5), highlighting an unresolved challenge in patient selection and pathway design [11]. These constraints motivate evaluation of outpatient neuromodulation strategies that can be positioned earlier within the care pathway.

Electrical pudendal nerve stimulation (EPNS) is a minimally invasive, low-cost, outpatient neuromodulation approach for lower urinary tract dysfunction and could potentially serve as a first-step entry within a tiered-care pathway before considering SNM [12], [13], [14]. Although previous reports suggest that EPNS produces improvements in bladder emptying and symptoms [15], [16], [17], existing evidence is limited by small sample sizes and a lack of direct comparison with the established SNM test stimulation strategy.

We conducted a prospective, comparative study directly comparing short-term outcomes of EPNS and SNM in adults with NOUR to assess the potential role of EPNS as an initial, nonimplantable neuromodulation option within a staged management pathway.

## 2 Materials and methods

### 2.1 Trial design and oversight

**Fig. 1:**
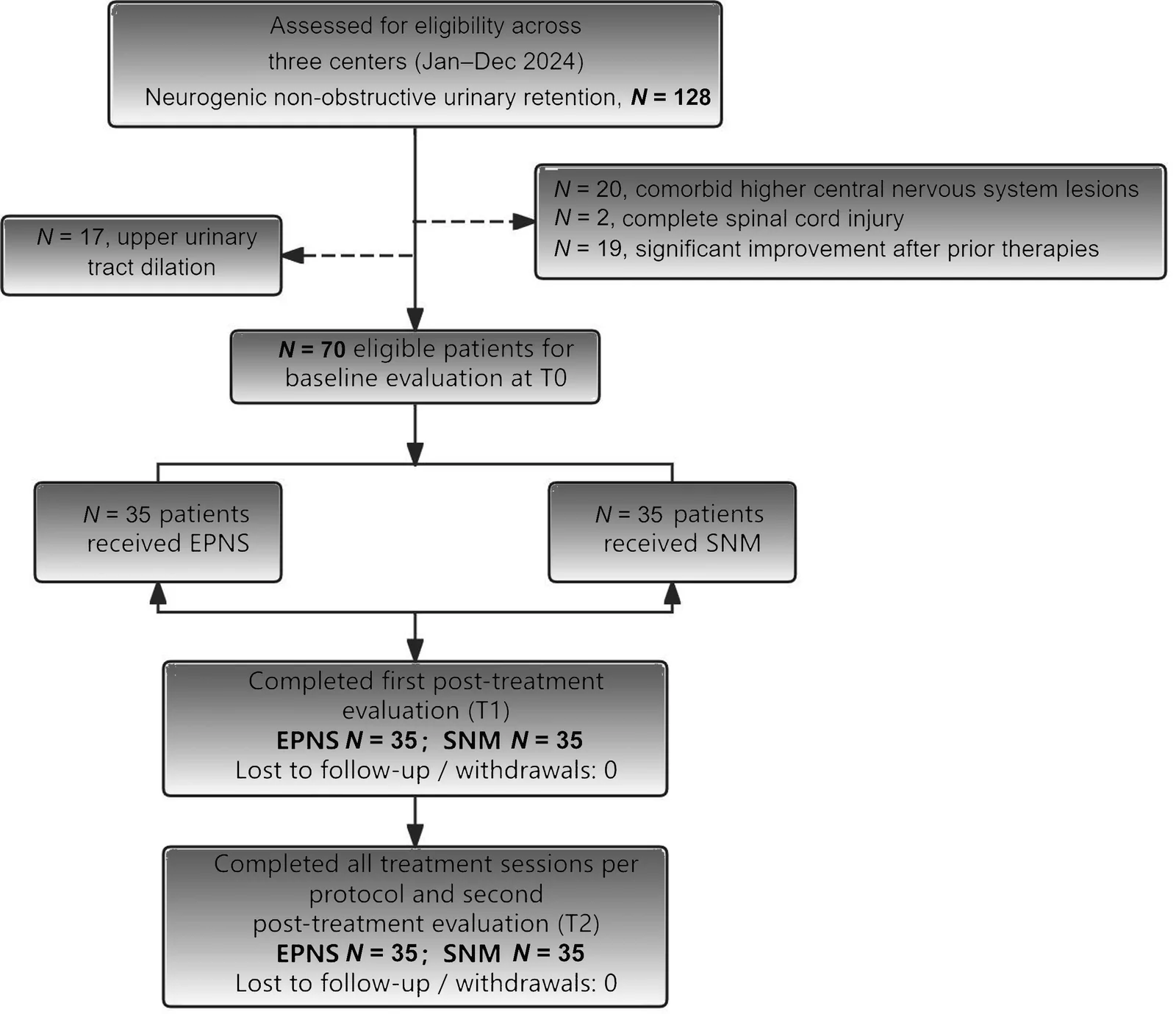
Patients’ flowchart. Across three centers, 128 adults with neurogenic nonobstructive urinary retention were assessed for eligibility. Fifty-eight were excluded: upper urinary tract dilation (hydronephrosis ± hydroureter), *n* = 17; comorbid higher central nervous system lesions, *n* = 20; complete spinal cord injury, *n* = 2; and significant improvement after previous therapies, *n* = 19. Seventy eligible patients completed T_0_ evaluation. All participants completed the first post-treatment evaluation (T_1_) and the second post-treatment evaluation (T_2_); no losses to follow-up or withdrawals occurred. EPNS = electrical pudendal nerve stimulation; SNM = sacral neuromodulation; T_0_ = baseline; T_1_ = after the sixth session of treatment; T_2_ = after the 12th session of treatment.

This was a prospective, multicenter, comparative study conducted at three hospitals in Shanghai from January 1 to December 31, 2024. The protocol was approved by the local ethics committee (no. 2018-099) and registered at ClinicalTrials.gov (NCT06158074). Because SNM involves device implantation and substantial out-of-pocket cost, random allocation was not feasible; therefore, treatment assignment followed patient preference within a shared decision-making process after standardized disclosure of procedures, risks, and costs. No allocation concealment was used. All participants provided a written informed consent. Outcome assessors were masked to treatment assignment. Reporting adhered to the Transparent Reporting of Evaluations with Nonrandomized Designs statement with a study flow diagram provided (Fig. 1).

### 2.2 Participants

Participants were consecutively enrolled at three sites. Eligible participants were adults aged 18–85 yr with chronic NOUR secondary to neurologic disease or injury, defined by symptom duration of ≥3 mo, absence of anatomic outlet obstruction, dependence on catheter-assisted bladder emptying (clean intermittent catheterization or an indwelling transurethral/suprapubic catheter), and a screening baseline (T_0_) PVR volume of ≥150 ml after an attempted void. Prespecified neurologic etiologies included suprasacral, sacral/infrasacral, and peripheral lesions (detailed in Supplementary Table 1). Additional eligibility criteria were preserved upper urinary tract and renal status (serum creatinine <1.2 mg/dL and no hydronephrosis on ultrasonography), discontinuation for ≥2 wk of therapies or medications that could affect lower urinary tract function, and the ability to adhere to treatment and follow-up. Key exclusion criteria were coexisting brainstem or supratentorial lesions, complete spinal cord injury, pregnancy or breastfeeding, mechanical outlet obstruction, active prostatitis, prostate cancer, other malignancy, and severe systemic disease. Participants could be withdrawn for protocol violations, nonadherence, serious adverse events (AEs), newly developed comorbidities, voluntary withdrawal, or death.

### 2.3 Interventions

Participants in the EPNS group received 12 sessions over 4 consecutive wk (3 sessions/wk) using a standardized, prespecified protocol. Each session was administered by the same licensed acupuncturist with the participant in the prone position after bladder emptying. Two upper points, located approximately 1 cm lateral to the sacrococcygeal joint, were needled perpendicularly (0.40 × 100 mm, depth 80–90 mm), and two lower points, located 1 cm lateral to the coccyx tip, were needled obliquely toward the ischiorectal fossa (0.40 × 125 mm, depth 90–110 mm). Each ipsilateral needle pair was connected to an electroacupuncture device, with the upper needle as anode and the lower as cathode. Electrical stimulation was delivered at 2.5 Hz for 60 min/session, with current intensity titrated within a prespecified range (25–35 mA) to elicit a tolerated urethral or perineal referred sensation. Treatment adherence was monitored prospectively. Detailed procedural information is provided in the Supplementary Methods 1.

Participants in the SNM group underwent a standardized 4-wk continuous test phase. Under local anesthesia, a quadripolar tined lead was placed in the S3 foramen using imaging-assisted localization and connected to an external stimulator. Correct lead placement was confirmed by characteristic motor and sensory responses to intraoperative stimulation, after which test stimulation was continued throughout the 4-wk period according to standard clinical programming. Additional procedural details are presented in the Supplementary Methods 2.

In both groups, stable medications for comorbidities were allowed, but no additional treatments affecting lower urinary tract function were permitted during the study.

### 2.4 Outcome measures

Independent masked assessors performed evaluations at T_0_, 2 wk (T_1_), and 4 wk (T_2_). The primary outcome was percentage change in PVR from T_0_, assessed at T_1_ and T_2_ as ([T_0_ PVR − follow-up PVR]/T_0_ PVR × 100%). Secondary continuous outcomes were percentage change from T_0_ in the International Consultation on Incontinence Questionnaire–Lower Urinary Tract Symptoms (ICIQ-LUTS) and Quality of Life (ICIQ-LUTSqol). Additional prespecified supportive binary endpoints at T_1_ and T_2_ were the achievement of PVR <50 ml, achievement of ≥50% PVR improvement, and a composite endpoint requiring both [18].

PVR was assessed after an attempted void using prespecified procedures. In participants performing clean intermittent catheterization, residual volume was measured by catheterization when feasible at the assessment visit and otherwise by bedside ultrasonography. In participants with an indwelling catheter, the catheter was temporarily clamped to allow an attempted void, after which residual volume was determined by catheter drainage. Threshold-related measurement uncertainty was addressed in prespecified robustness analyses (Section 2.6).

### 2.5 Sample size calculation

Sample size was estimated for the primary outcome using internal preliminary data showing mean (standard deviation [SD]) PVR improvement rate of 65% (25%) for EPNS and 47% (19%) for SNM. Using a 2-sided α of 0.05 and 90% power, 28 participants per group were required by a two-sample *t* test. Allowing for 10% attrition, the planned sample size was 32 per group; 35 per group (70 total) were ultimately enrolled to allow for unforeseen data loss.

### 2.6 Statistical analysis

All analyses were 2-sided with α = 0.05 and performed in SPSS version 31.0 (IBM, Armonk, NY, USA). All enrolled participants completed follow-up assessments at T_1_ and T_2_; no imputation was required.

Continuous outcomes at T_1_ and T_2_ were analyzed using linear mixed-effects models (LMM) with a subject-level random intercept and an autoregressive covariance structure of order 1. Fixed effects were treatment group, time, and the group-by-time interaction, with adjustment for sex, age, duration, lesion pattern, T_0_ value of PVR, and questionnaire scores. For each continuous outcome, covariate-adjusted least-squares means were obtained for each group-by-time cell from the longitudinal LMM including both post-T_0_ assessments, rather than from unadjusted summaries or separate time-specific models. We report the omnibus type III test for the group-by-time interaction and prespecified Bonferroni-adjusted between-group contrasts at T_1_ and T_2_ from the same model as estimated mean differences with 95% confidence intervals (CIs) (percentage points for improvement-rate outcomes). As a sensitivity analysis, untransformed absolute PVR volumes at T_1_ and T_2_ were analyzed as the dependent variable using the same adjusted LMM, with T_0_ PVR included as a covariate.

Binary endpoints at T_1_ and T_2_ were compared using two-sided Fisher’s exact tests without across-outcome multiplicity control.

In the primary analyses, PVR was dealt on the raw scale (0 ml denoting complete emptying for computing absolute change). To address threshold-related uncertainty in PVR-based outcomes, prespecified robustness analyses used anchors of ≤40, ≤50, and ≤60 ml. For each anchor, values at or below the threshold were set to 0 and values above the threshold were expressed as the excess above that threshold; PVR-based continuous and binary endpoints were then recomputed using the same analytic framework. Additional robustness analyses for continuous outcomes at T_2_ used multivariable linear regression in the crude cohort and in a 1:1 nearest-neighbor propensity-score–matched (PSM) cohort without replacement (caliper = 0.2 SD of the logit of the propensity score). In the crude cohort, model 1 was adjusted for sex, age, symptom duration, and lesion pattern, and model 2 was further adjusted for T_0_ values of PVR and questionnaire scores. For binary endpoints at T_2_, E-values quantified the unmeasured confounding needed to explain away observed associations.

### 2.7 Safety monitoring

AEs were prospectively collected and graded according to the Common Terminology Criteria for Adverse Events (version 5.0) and were assessed for attribution to the assigned intervention. Prespecified procedure- or device-related events included lead-site soreness and infection. Serious AEs were adjudicated by an independent physician.

## 3 Results

### 3.1 Study population and T_0_ characteristics

Seventy participants were enrolled, with 35 in each group. All completed assessments at T_0_, T_1_, and T_2_, and no outcome data were missing. T_0_ characteristics for the crude and PSM cohorts are presented in Table 1. Before matching, several covariates differed between groups. After 1:1 PSM, 24 pairs (*n* = 48) were retained, with improved covariate balance overall; minor residual imbalance remained for symptom duration and T_0_ questionnaire scores.

**Table 1:** Baseline characteristics between groups in crude and matched cohorts

Variables	Crude cohort			Matched cohort^a^		
	EPNS (*n* = 35)	SNM (*n* = 35)	|SMD|^b^	EPNS (*n* = 24)	SNM (*n* = 24)	|SMD|^b^
Age, median (Q_1_, Q_3_), y	50 (42, 62)	54 (45, 65)	0.28	52 (45, 63)	52 (42, 60)	0.01
Duration, median (Q_1_, Q_3_), m	9 (5, 23)	14 (7, 36)	0.03	9 (5, 42)	11 (6, 36)	0.21
Sex, n (%)						
Male	15 (43)	17 (49)	0.11	12 (50)	12 (50)	0.00
Lesion pattern, n (%)^c^						
Suprasacral spinal lesion	9 (26)	14 (40)	0.29	8 (33)	9 (37)	0.09
PVR_0_, median (Q_1_, Q_3_), ml	200 (150, 400)	260 (181, 332)	0.17	204 (150, 385)	249 (155, 309)	0.09
ICIQ-LUTS_0_, median (Q_1_, Q_3_)	24 (16, 32)	22 (17, 28)	0.22	23 (16, 32)	24 (16, 28)	0.14
ICIQ-LUTSqol_0_, median (Q_1_, Q_3_)	50 (40,57)	52 (48, 56)	0.36	53 (49, 58)	52 (49, 58)	0.13

EPNS = electrical pudendal nerve stimulation; ICIQ-LUTS = International Consultation on Incontinence Questionnaire–Lower Urinary Tract Symptoms; ICIQ-LUTSqol = International Consultation on Incontinence Questionnaire–Lower Urinary Tract Symptoms Quality of Life; PVR = postvoid residual; Q_1_ = first quartile; Q_3_ = third quartile; SMD = standardized mean difference; SNM = sacral neuromodulation.

a The matched cohort was derived using 1:1 nearest-neighbor propensity score matching without replacement, with a caliper of 0.2 standard deviation of the logit of the propensity score. Propensity scores were estimated using logistic regression including age, sex, duration, lesion pattern, and baseline PVR_0_, ICIQ-LUTS_0_, and ICIQ-LUTSqol_0_. Covariate balance was considered adequate if the absolute |SMD| was <0.10. The process yielded 24 matched pairs (*n* = 48), with 22 participants remaining unmatched.

b For continuous variables displayed as median (Q_1_, Q_3_,), SMDs were calculated on the original continuous scale; for categorical variables, SMDs are for proportions.

c The complementary category included sacral/infrasacral, peripheral pelvic nerve, and other specified nonsuprasacral neurologic disorders, as presented in Supplementary Table 1. Supratentorial and brainstem lesions were excluded by the eligibility criteria.

### 3.2 Primary outcome and key secondary continuous outcomes

For the primary endpoint, EPNS outperformed SNM at both follow-up points in the longitudinal LMM for percentage PVR improvement. At T_2_, adjusted least-squares mean PVR improvement rates were 68% for EPNS and 51% for SNM, corresponding to a between-group difference of 17 percentage points (95% CI 5–29; *p* = 0.008). A similar difference was observed at T_1_ (18 percentage points; 95% CI 5–30; *p* = 0.005). The group-by-time interaction was not significant (*p* for interaction = 0.8).

**Fig. 2:**
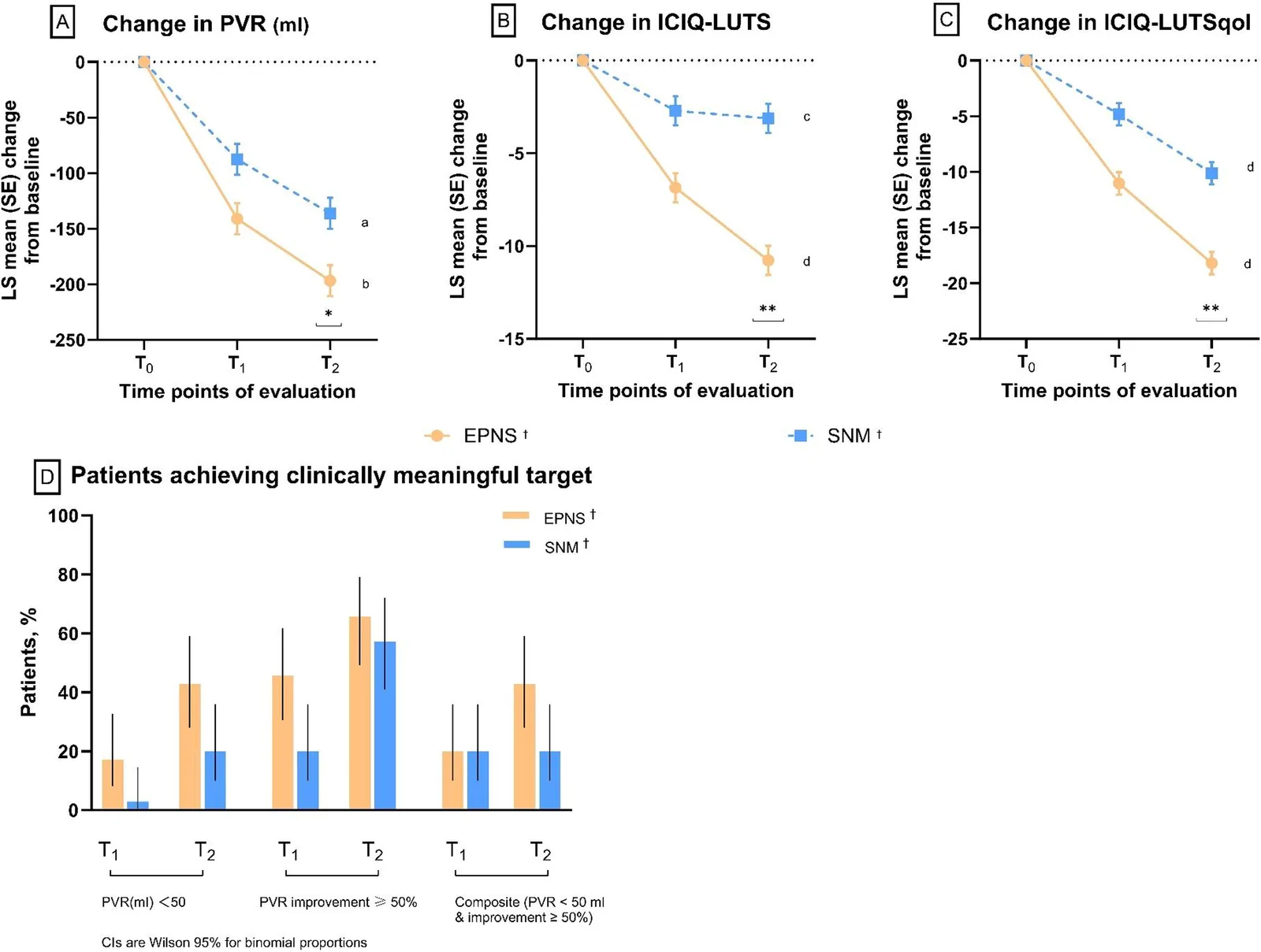
Longitudinal changes in PVR, ICIQ-LUTS, and ICIQ-LUTSqol by treatment group and proportions meeting prespecified targets. (A–C) Lines show least-squares means (LS-means) from linear mixed-effects models for repeated measures. Fixed effects were treatment groups, time, and their interaction; models used a random intercept with first-order autoregressive within-subject covariance and were adjusted for sex, age, symptom duration, lesion pattern, and the baseline value of the corresponding outcome. Error bars indicate standard errors of the LS-means. (D) Bars show observed proportions for the predefined targets at T_1_ and T_2_; values are descriptive. Error bars indicate Wilson 95% confidence intervals for binomial proportions. “*” and “**” indicate between-group comparisons at the same time point with Bonferroni adjustment (*p = 0.008; **p < 0.001). Letters indicate within-group pairwise comparisons between time points with Bonferroni adjustment (a, *p = 0.007; b, *p = 0.001; c, *p = 01.00; d, *p* < 0.001). “†” indicates sample size: *n* = 35 per group per time point (applies to panels A–D). EPNS = electrical pudendal nerve stimulation; ICIQ-LUTS = International Consultation on Incontinence Questionnaire–Lower Urinary Tract Symptoms; ICIQ-LUTSqol = International Consultation on Incontinence Questionnaire–Lower Urinary Tract Symptoms Quality of Life; PVR = postvoid residual; SNM = sacral neuromodulation; T_0_ = baseline; T_1_ = after the sixth session of treatment; T_2_ = after the 12th session of treatment.

For key secondary outcomes, greater improvements were also observed in the EPNS group. At T_2_, the adjusted between-group difference was 28 percentage points (95% CI 17–39; *p* < 0.001) for ICIQ-LUTS and 17 percentage points (95% CI 10–24; *p* < 0.001) for ICIQ-LUTSqol. T_1_ results were similar (Table 2A). The group-by-time interaction was significant for ICIQ-LUTS (*p* < 0.001) but not for ICIQ-LUTSqol (*p* = 0.14). Longitudinal trajectories on the raw scales are presented in Figure 2A–2C, with visit-specific estimates presented in Supplementary Table 2. Results for PVR-based continuous outcomes were directionally consistent in the threshold-robustness analyses using anchors of ≤40, ≤50, and ≤60 ml.

**Table 2:** Primary and key secondary outcomes at T_1_ and T_2_ (EPNS vs SNM)

A. Continuous outcomes						
Outcome		Time points	EPNS: LS-mean	SNM: LS-mean	Δ (EPNS–SNM), pp (95% CI)	*p* value
PVR improvement rate (%)		T_1_	50%	32%	18 pp (5–30)	0.005
		T_2_	68%	51%	17 pp (5–29)	0.008
ICIQ-LUTS improvement rate (%)		T_1_	26%	14%	12 pp (2–23)	0.03
		T_2_	43%	15%	28 pp (17–39)	<0.001
ICIQ-LUTSqol improvement rate (%)		T_1_	23%	9%	14 pp (7–20)	<0.001
		T_2_	36%	19%	17 pp (10–24)	<0.001

**B. Binary target outcomes**						

**Target**	**Time points**	**EPNS: n/N, risk %**	**SNM: n/N, risk %**	**RD, pp (95% CI)**	**RR (95% CI)**	***p* value (Fisher)**

PVR <50 ml	T_1_	7/35, 20%	0/35, 0%	20 (0–36)	15 (1–253)	0.01
	T_2_	15/35, 43%	7/35, 20%	23 (−8 to 49)	2 (1–5)	0.07
PVR improvement ≥50%	T_1_	16/35, 46%	7/35, 20%	26 (−5 to 52)	2 (1–5)	0.04
	T_2_	23/35, 66%	20/35, 57%	9 (−23 to 38)	1 (1–2)	0.6
Composite	T_1_	7/35, 20%	0/35, 0 %	20 (0–36)	15 (1–253)	0.01
	T_2_	15/35, 43%	7/35, 20%	23 (−8 to 49)	2 (1–5)	0.07

CI = confidence interval; EPNS = electrical pudendal nerve stimulation; ICIQ-LUTS = International Consultation on Incontinence Questionnaire–Lower Urinary Tract Symptoms; ICIQ-LUTSqol = ICIQ-LUTS quality of life; LS-mean = least-squares mean; pp = percentage points; PVR = postvoid residual; RD = risk difference; RR = relative risk; SNM = sacral neuromodulation; T_1_ = after the sixth session of treatment; T_2_ = after the 12th session of treatment.

Panel A (continuous outcomes): Values are covariate-adjusted LS-means from longitudinal linear mixed-effects models including both postbaseline assessments at T_1_ and T_2_. Models included fixed effects for treatment group, time, and the group-by-time interaction and were adjusted for sex, age, symptom duration, lesion pattern, baseline PVR, baseline ICIQ-LUTS, and baseline ICIQ-LUTSqol. Between-group differences are model-based contrasts from the same models and are reported with 95% CIs; contrasts at T_1_ and T_2_ were Bonferroni-adjusted within outcome. Panel B (binary targets): Risks are shown as observed n/N and percentages. RD is reported with Newcombe 95% CIs; RR is reported with 95% CIs using the Haldane–Anscombe correction when applicable. *p* values are from two-sided Fisher’s exact tests. Denominators are observed cases; no imputation was used.

### 3.3 Prespecified binary clinical targets

Response rates generally favored EPNS (Fig. 2D, Table 2B). At T_1_, ≥50% PVR improvement occurred in 46% versus 20% (*p* = 0.04). At T_2_, rates were 66% versus 57% with no significant between-group differences (relative risk 1; 95% CI 1–2; *p* = 0.6). For the PVR <50 ml and composite targets, the between-group pattern was consistent across time points. At T_2_, response rates for the PVR <50 ml target were 43% versus 20% (relative risk 2; 95% CI 1–5; *p* = 0.07), and findings were identical for the composite target. Threshold-adjusted analyses were directionally consistent with reduced precision (Supplementary Table 3).

### 3.4 Robustness and sensitivity analyses

In multivariable linear regression, the primary endpoint favored EPNS in the crude cohort (model 2 β −0.16; 95% CI −0.28 to −0.03; *p* = 0.02); in the matched cohort, the point estimate was in the same direction, but the CI included the null value (β −0.13; 95% CI −0.27 to 0.01; *p* = 0.08). Questionnaire scores were consistent across analytic approaches (ICIQ-LUTS, model 2 β −0.28; *p* < 0.001; ICIQ-LUTSqol, model 2 β −0.17; *p* < 0.001), with concordant results in the matched cohort (Table 3). In threshold-adjusted datasets, effect estimates were similar in direction and magnitude in both crude and matched cohorts (Supplementary Table 4). In an additional sensitivity analysis using untransformed absolute PVR volume as the dependent variable in the adjusted LMM, EPNS showed lower adjusted PVR than SNM at T_1_ (137 vs 180 ml; difference −43 ml; 95% CI −82 to −5; *p* = 0.03) and T_2_ (81 vs 132 ml; difference −50 ml; 95% CI −89 to −12; *p* = 0.01) (Supplementary Table 5).

**Table 3:** Multivariable regression models for primary and key secondary outcomes (robustness checks)

Outcome	Crude cohort—model 1	*p* value	Crude cohort—model 2	*p* value	Matched cohort—model 1	*p* value
	β (95% CI)		β (95% CI)		β (95% CI)	
PVR improvement rate	−0.14 (−0.26 to −0.03)	0.02	−0.16 (−0.28 to −0.03)	0.02	−0.13 (−0.27 to 0.01)	0.08
ICIQ-LUTS improvement rate	−0.29 (−0.40 to −0.18)	<0.001	−0.28 (−0.40 to −0.17)	<0.001	−0.30 (−0.43 to −0.17)	<0.001
ICIQ-LUTSqol improvement rate	−0.16 (−0.23 to −0.09)	<0.001	−0.17 (−0.25 to −0.10)	<0.001	−0.17 (−0.25 to −0.08)	<0.001

CI = confidence interval; EPNS = electrical pudendal nerve stimulation; ICIQ-LUTS = International Consultation on Incontinence Questionnaire–Lower Urinary Tract Symptoms; ICIQ-LUTSqol = International Consultation on Incontinence Questionnaire–Lower Urinary Tract Symptoms Quality of Life; PVR = postvoid residual; SNM = sacral neuromodulation.

β: β values are on the proportion scale for improvement-rate outcomes and represent the effect of SNM compared with EPNS (reference group); negative β indicates less improvement with SNM than with EPNS. Sample: crude cohort *n* = 35; matched cohort *n* = 24; the matched cohort was derived by propensity-score matching. Model 1 was adjusted for sex, age, duration, and lesion pattern. Model 2 was additionally adjusted for baseline severity (PVR_0_, ICIQ-LUTS_0_, and ICIQ-LUTSqol_0_).

For binary endpoints at T_2_, the E-value was 3.70 for both PVR <50 ml and the composite endpoint and 1.57 for ≥50% PVR improvement; lower-bound E-values were close to 1, indicating limited robustness at the confidence-limit boundary.

### 3.5 Safety and adherence

No serious AEs occurred. Transient perineal discomfort was reported in 3 EPNS participants, and lead-site soreness in 27 SNM participants. No AE-related withdrawals occurred. Protocol adherence was 100% in both groups.

## 4 Discussion

Our study suggests that a 12-session outpatient EPNS regimen was associated with greater short-term improvement than the SNM test phase, particularly in the prespecified primary endpoint and questionnaire scores. At the end of treatment, the adjusted PVR improvement rate was 68% with EPNS versus 51% with SNM (between-group difference 17 percentage points; 95% CI 5–29; *p* = 0.008), with parallel advantages in questionnaire-based outcomes. Similar directional findings were observed across follow-up assessments and sensitivity analyses.

In current practice, SNM is a guideline-recommended option for refractory NOUR, but it requires device implantation and periprocedural management. Against this background, EPNS offers a 4-wk outpatient course aligned with the SNM test phase and may serve as a less invasive step within a staged neuromodulation pathway. Viewed through a value-based health care lens [19], this preimplant step may help inform treatment escalation on the basis of initial treatment response in refractory NOUR. Whether early improvement translates into sustained symptom control remains uncertain and requires randomized studies with longer follow-up.

SNM was selected as the comparator because it represents an established neuromodulation pathway in current practice for refractory NOUR. The response rates observed in our SNM arm were broadly consistent with previous reports [20], [21], supporting the clinical relevance of this active comparator. By comparing EPNS with the SNM test phase rather than postimplant outcomes alone, the present design addresses a clinically important preimplant decision point and avoids the enrichment inherent in postimplant-only cohorts. Against this active standard, EPNS was associated with greater short-term improvement, supporting its potential role before implantation. Previous reports in SNM nonresponders further suggest that pudendal nerve stimulation may retain benefit even after inadequate SNM response, underscoring the clinical relevance of the pudendal pathway in refractory NOUR [16], [22].

Differences in neural interface and pudendal-somatic pathway engagement may partly underlie these findings. SNM acts through stimulation of the mixed S3 root (contributing a small part of the overall pudendal afferent activity) and is thought to modulate lower urinary tract function through afferent-mediated effects on sacral reflexes, pelvic floor activity, and higher voiding-control pathways [23]. This proximal access can influence bladder–outlet coordination, but does so using a mixed sacral-root interface [4]. By contrast, the EPNS protocol in this study delivers electrical stimulation through deeply inserted needles in the sacrococcygeal and coccygeal regions, with sensations referred to the urethral or anal region serving as procedural indicators of engagement of pudendal sensory territories (Supplementary Methods 1). This paradigm may provide a distal, pudendal-biased neural interface that preferentially recruits somatic afferent input from urethral and perineal territories (contributing a large part of the overall pudendal afferent activity) and may influence external urethral sphincter (EUS) activity through pudendal–sacral reflex pathways [24], [25], [26]. Within these pathways, pudendal afferents enter the sacral cord and interact with interneuronal circuits that integrate pudendal-somatic input with pelvic visceral afferent signaling, thereby modulating bladder afferent processing (including awareness of bladder filling), as well as urethral guarding reflexes and detrusor–outlet coordination [27], [28], [29]. In NOUR, where voiding failure may involve impaired detrusor activation, excessive outlet guarding, or detrusor–sphincter discoordination, such pudendal-biased modulation could facilitate bladder emptying by recalibrating urethral guarding and improving bladder–outlet coupling [30]. Although these mechanisms remain inferential in the present study, they provide biological plausibility for the observed short-term effects of EPNS.

This study has several limitations. First, its nonrandomized design leaves room for residual confounding and selection bias. Because SNM required out-of-pocket payment and socioeconomic/access variables were unavailable, affordability-related bias cannot be excluded despite matching; therefore, the findings should be considered hypothesis generating. Second, the modest sample and short follow-up limited precision and precluded subgroup analyses. Third, treatment parameters were fixed during a protocolized test period and may not reflect longer-term individualized optimization. Fourth, intermittent outpatient EPNS and continuous SNM test stimulation differed in delivery format and may have influenced comparative effects. Randomized studies with longer follow-up and mechanistic endpoints are needed.

## 5 Conclusion

In refractory NOUR, EPNS was associated with greater short-term improvement than the SNM test phase in both objective and questionnaire-based outcomes. These findings support consideration of EPNS as a potential initial outpatient step within a staged neuromodulation pathway.

  ***Author contributions:*** Siyou Wang had full access to all the data in the study and takes responsibility for the integrity of the data and the accuracy of the data analysis.

  *Study concept and design*: Wang and Lv.

*Acquisition of data*: Xu and Li.

*Analysis and interpretation of data*: Li.

*Drafting of the manuscript*: Li.

*Critical revision of the manuscript for important intellectual content*: Li, Xu, Lv, Huang and Wang.

*Statistical analysis*: Li.

*Obtaining funding*: Li and Huang.

*Administrative, technical, or material support*: Lv, Huang and Wang.

*Supervision*: Huang and Wang.

*Project administration:* Wang and Lv.

  ***Financial disclosures:*** Wang certifies that all conflicts of interest, including specific financial interests and relationships and affiliations relevant to the subject matter or materials discussed in the manuscript (eg, employment/affiliation, grants or funding, consultancies, honoraria, stock ownership or options, expert testimony, royalties, or patents filed, received, or pending), are the following: None.

  ***Funding/Support and role of the sponsor:*** The study is supported by Shanghai Pudong New Area Health Commission (The Medical Discipline Construction Program of Shanghai Pudong New Area Health Commission-the Specialty Program PWZzb2025-16) and Shanghai Pudong New Area Health Commission (New Quality Clinical Specialty Program of High-end Medical Disciplinary Construction in Shanghai Pudong New Area 2025-PWXZ-17).

  ***Ethics approval and consent to participate:*** The study was approved by the local institutional review board following the Declaration of Helsinki (no. 2018-099). All participants provided a written informed consent.

  ***Availability of data and materials:*** Deidentified participant data will be available upon publication. Requests should be sent to the corresponding author with a valid methodology. Approved researchers with a signed data access agreement will have access to the data.

## Appendix A Supplementary data

Supplementary Data 1 Additional tables and methodological details, including etiological categories, adjusted outcome estimates, sensitivity analyses, and standardized EPNS and SNM protocols.
