# Supplementary material for: Electrical Pudendal Nerve Stimulation as an Initial Neuromodulation Strategy for Neurogenic Urinary Retention
Source: Eur Urol Open Sci. 2026 Sep 4;92:19–27. doi: 10.1016/j.euros.2026.07.008 (PMC13571503; doi:10.1016/j.euros.2026.07.008)
Supplement: Supplementary file 1 [file mmc1.docx]

| **Supplementary table 1. Etiological categories of neurogenic disorders in both groups** | | | | |
| --- | --- | --- | --- | --- |
| Lesion level | Mechanism / Primary diagnosis | Representative examples in this cohort | EPNS group  (n=35) | SNM group (n=35) |
| Suprasacral (cervical/thoracic cord) | Traumatic spinal cord injury | Traumatic spinal cord injury at cervical/thoracic levels | 5 | 8 |
|  | Inflammatory/demyelinating | Myelitis involving cervical/thoracic cord | 3 | 2 |
|  | Cavitary/structural | Syringomyelia at thoracic or cervical levels | 1 | 2 |
|  | Hemorrhagic/vascular | Intramedullary hematoma | 0 | 2 |
|  |  |  |  |  |
| Sacral / Infrasacral (conus medullaris / cauda equina) | Structural | Tethered cord syndrome | 3 | 2 |
|  | Compressive–degenerative | Lumbar disc disease with cauda equina syndrome | 3 | 2 |
|  | Neoplastic | Lumbosacral (conus/cauda equina) tumors | 2 | 2 |
|  |  |  |  |  |
| Peripheral pelvic nerve | Iatrogenic pelvic surgery–related neuropathy | Gynecologic pelvic surgery (e.g., radical hysterectomy for cervical carcinoma; hysterectomy) | 12 | 8 |
|  |  |  |  |  |
| Other specified neurological disorders | Other neurological diseases | Multiple sclerosis, diabetic cystopathy, etc. | 6 | 7 |

Etiologies were classified hierarchically by lesion level (Suprasacral vs sacral/infrasacral vs peripheral) and then by mechanism. Each patient was assigned one primary etiology. Surgical procedures are listed as iatrogenic mechanisms or examples rather than standalone etiologies.

| **Supplementary table 2. Adjusted mean values of key outcome measures at each time point and between-group differences at T_2_, based on linear mixed-effects models** | | | | | | | | |
| --- | --- | --- | --- | --- | --- | --- | --- | --- |
| Outcome |  | Group | T_0_ | T_1_ | T_2_ | Change from baseline (T_2_–T_0_) | Between-group difference at T_2_ (95% CI) | *P* |
| PVR (mL) | Threshold=0 | EPNS | 276 | 135 | 80 | -197 | -54 (-93 to -14) | 0.008 |
|  |  | SNM | 233 | 181 | 133 | -100 |  |  |
|  |  |  |  |  |  |  |  |  |
|  | Threshold=40 | EPNS | 237 | 100 | 50 | -186 | -43 (-82 to -5) | 0.03 |
|  |  | SNM | 229 | 141 | 94 | -135 |  |  |
|  |  |  |  |  |  |  |  |  |
|  | Threshold=50 | EPNS | 223 | 92 | 44 | -182 | -41 (-79 to -2) | 0.045 |
|  |  | SNM | 219 | 131 | 85 | -134 |  |  |
|  |  |  |  |  |  |  |  |  |
|  | Threshold=60 | EPNS | 217 | 85 | 39 | -178 | -38 (-76 to 0.2) | 0.051 |
|  |  | SNM | 209 | 122 | 77 | -132 |  |  |
|  |  |  |  |  |  |  |  |  |
|  |  |  |  |  |  |  |  |  |
| ICIQ-LUTS |  | EPNS | 24 | 17 | 13 | -11 | -7 (-10 to -5) | <0.001 |
|  |  | SNM | 24 | 20 | 21 | -3 |  |  |
|  |  |  |  |  |  |  |  |  |
| ICIQ-LUTSqol |  | EPNS | 50 | 39 | 32 | -18 | -9 (-12 to -6) | <0.001 |
|  |  | SNM | 51 | 46 | 41 | -10 |  |  |
| Abbreviation; EPNS, Electrical Pudendal Nerve Stimulation; SNM, Sacral Neuromodulation; PVR, Postvoid Residual; ICIQ-LUTS, International Consultation on Incontinence Questionnaire–Lower Urinary Tract Symptoms; ICIQ-LUTSqol, International Consultation on Incontinence Questionnaire–Lower Urinary Tract Symptoms Quality of Life; T_0_, baseline; T_1_, after the 6^th^ sessions of treatment; T_2_, after the 12^th^ sessions of treatment. | | | | | | | | |
| Values are adjusted least-squares means from linear mixed-effects models (random intercept; AR[1]); covariates: sex, age, symptom duration, lesion pattern, and baseline value of the outcome. For the threshold-adjusted PVR rows, we assumed values at or below the stated threshold (40/50/60 mL) are physiologically normal and subtracted the threshold from each measured PVR at T_0_, T_1_, and T_2_; values at or below the threshold were recorded as 0, and values above the threshold were recorded as the measured value minus the threshold. All PVR-based metrics in those rows were recomputed accordingly. ICIQ-LUTS and ICIQ-LUTSqol do not depend on the PVR threshold and are reported once for clarity.  Between-group difference at T_2_ is EPNS minus SNM; negative values indicate greater improvement in EPNS. The purpose of including T=0/40/50/60 side-by-side is sensitivity analysis, not multiple independent hypothesis testing. | | | | | | | | |

| **Supplementary table 3-1. Primary and key secondary outcomes at T_1_ and T_2_ (physiologically normal defined as PVR ≤ 40 mL)** | | | | | |
| --- | --- | --- | --- | --- | --- |
| A. Continuous outcomes | | | | | |
| Outcome | Time points | EPNS: LS-mean | SNM: LS-mean | Δ (EPNS–SNM), pp (95% CI) | *P* |
|  |  |  |  |  |  |
| PVR improvement rate (%) | T_1_ | 59 | 40 | 19 (5 to 32) | 0.009 |
|  | T_2_ | 77 | 63 | 15 (1 to 28) | 0.04 |
|  |  |  |  |  |  |
| ICIQ-LUTS improvement rate (%) | T_1_ | 26 | 14 | 12 (2 to 23) | 0.02 |
|  | T_2_ | 43 | 15 | 28 (17 to 39) | <0.001 |
|  |  |  |  |  |  |
| ICIQ-LUTSqol improvement rate (%) | T_1_ | 23 | 9 | 14 (7 to 20) | <0.001 |
|  | T_2_ | 36 | 19 | 17 (10 to 24) | <0.001 |

| B. Binary target outcomes | | | | | | |
| --- | --- | --- | --- | --- | --- | --- |
| Target | Time points | EPNS: n/N，  risk % | SNM: n/N，  risk % | RD, %  (Newcombe 95% CI) | RR  (95% CI) | *P* (Fisher) |
|  |  |  |  |  |  |  |
| PVR<40mL | T_1_ | 7/35, 20 | 0/35, 0 | 20 (0 to 36) | 15 (1 to 253) | 0.01 |
|  | T_2_ | 12/35, 34 | 3/35, 9 | 26 (-2 to 48) | 4 (1 to13) | 0.02 |
|  |  |  |  |  |  |  |
| PVR improvement≥50% | T_1_ | 18/35, 51 | 12/35,34 | 17 (-15 to 46) | 2 (1 to 3) | 0.2 |
|  | T_2_ | 25/35, 71 | 23/35, 66 | 6 (-24 to 35) | 1 (1 to 2) | 0.8 |
|  |  |  |  |  |  |  |
| Composite | T_1_ | 7/35, 20 | 0/35, 0 | 20 (0 to 36) | 15 (1 to 253) | 0.01 |
|  | T_2_ | 12/35, 34 | 3/35, 9 | 26 (-2 to 48) | 4 (1 to13) | 0.02 |
| Abbreviations: EPNS, electrical pudendal nerve stimulation; SNM, sacral neuromodulation; LS-mean, least-squares mean; RD, risk difference; RR, relative risk; CI, confidence interval; PVR, post-void residual; ICIQ-LUTS, International Consultation on Incontinence Questionnaire–Lower Urinary Tract Symptoms; ICIQ-LUTSqol, ICIQ-LUTS quality of life.  Assuming PVR ≤40 mL is physiologically normal, we redefined PVR at all time points by subtracting 40 mL from each measured value; values ≤40 mL were recorded as 0, and values >40 mL were recorded as the measured value minus 40 mL. All PVR-based endpoints were recomputed accordingly. “Composite” denotes meeting both “PVR ≤40 mL” and “PVR improvement rate ≥50%” from baseline. | | | | | | |
| Panel A (continuous outcomes): Values are covariate-adjusted LS-means from linear mixed-effects models (random intercept; AR[1]); covariates: sex, age, symptom duration, lesion pattern, and baseline value of the corresponding outcome. Between-group contrasts at T_1_ and T_2_ are Bonferroni-adjusted within outcome. pp = percentage points. | | | | | | |
| Panel B (binary targets): Risks are shown as observed n/N and percentages. RD is reported with Newcombe 95% CIs; RR is reported with 95% CIs using the Haldane–Anscombe correction when applicable. P values are from two-sided Fisher’s exact tests. Denominators are observed cases; no imputation was used. | | | | | | |

| **Supplementary table 3-2. Primary and key secondary outcomes at T_1_ and T_2_ (physiologically normal defined as PVR ≤ 50 mL)** | | | | | | | | | | | |  |
| --- | --- | --- | --- | --- | --- | --- | --- | --- | --- | --- | --- | --- |
| A. Continuous outcomes | | | | | | | | | | | |  |
| Outcome | | | Time points | EPNS: LS-mean | | SNM: LS-mean | | Δ (EPNS–SNM), pp (95% CI) | | *P* | |  |
|  | | |  |  | |  | |  | |  | |  |
| PVR improvement rate (%) | | | T_1_ | 61 | | 43 | | 18 (4 to 32) | | 0.01 | |  |
|  |  |  | T_2_ | 80 | | 66 | | 14 (1 to 28) | | 0.06 | |  |
|  | | |  |  | |  | |  | |  | |  |
| ICIQ-LUTS improvement rate (%) | | | T_1_ | 26 | | 14 | | 12 (2 to 23) | | 0.03 | |  |
|  |  |  | T_2_ | 43 | | 15 | | 28 (17 to 39) | | <0.001 | |  |
|  | | |  |  | |  | |  | |  | |  |
| ICIQ-LUTSqol improvement rate (%) | | | T_1_ | 23 | | 9 | | 14 (7 to 20) | | <0.001 | |  |
|  |  |  | T_2_ | 36 | | 19 | | 17 (10 to 24) | | <0.001 | |  |
| B. Binary target outcomes | | | | | | | | | | | | |
| Target | Time points | EPNS: n/N，  risk % | | | SNM: n/N，  risk % | | RD, %  (Newcombe 95% CI) | | RR  (95% CI) | | *P* (Fisher) | |
|  |  |  | | |  | |  | |  | |  | |
| PVR<50mL | T_1_ | 8/35, 23 | | | 0/35, 0 | | 23 (2 to 39) | | 17 (1 to 284) | | 0.005 | |
|  | T_2_ | 15/35, 43 | | | 7/35, 20 | | 23 (-8 to 49) | | 2 (1 to 4) | | 0.07 | |
|  |  |  | | |  | |  | |  | |  | |
| PVR improvement≥50% | T_1_ | 19/35, 54 | | | 15/35, 49 | | 11 (-21 to 42) | | 1 (1 to 2) | | 0.47 | |
|  | T_2_ | 28/35, 80 | | | 23/35, 65 | | 14 (-15 to 41) | | 1 (1 to 2) | | 0.3 | |
|  |  |  | | |  | |  | |  | |  | |
| Composite | T_1_ | 8/35, 23 | | | 0/35, 0 | | 23 (2 to 39) | | 17 (1 to 284) | | 0.005 | |
|  | T_2_ | 15/35, 43 | | | 7/35, 20 | | 23 (-8 to 49) | | 2 (1 to 4) | | 0.07 | |
| Abbreviations: EPNS, electrical pudendal nerve stimulation; SNM, sacral neuromodulation; LS-mean, least-squares mean; RD, risk difference; RR, relative risk; CI, confidence interval; PVR, post-void residual; ICIQ-LUTS, International Consultation on Incontinence Questionnaire–Lower Urinary Tract Symptoms; ICIQ-LUTSqol, ICIQ-LUTS quality of life.  Assuming PVR ≤50 mL is physiologically normal, we subtracted 50 mL from each PVR measurement at all time points. Values ≤50 mL were recorded as 0, and values >50 mL were recorded as the measured value minus 50 mL. All PVR-based endpoints were recomputed under this definition. “Composite” denotes meeting both “PVR ≤50 mL” and “PVR improvement rate ≥50%” from baseline. | | | | | | | | | | | | |
| Panel A (continuous outcomes): Values are covariate-adjusted LS-means from linear mixed-effects models (random intercept; AR[1]); covariates: sex, age, symptom duration, lesion pattern, and baseline value of the corresponding outcome. Between-group contrasts at T_1_ and T_2_ are Bonferroni-adjusted within outcome. pp = percentage points. | | | | | | | | | | | | |
| Panel B (binary targets): Risks are shown as observed n/N and percentages. RD is reported with Newcombe 95% CIs; RR is reported with 95% CIs using the Haldane–Anscombe correction when applicable. P values are from two-sided Fisher’s exact tests. Denominators are observed cases; no imputation was used. | | | | | | | | | | | | |

| **Supplementary table 3-3. Primary and key secondary outcomes at T_1_ and T_2_ (physiologically normal defined as PVR ≤ 60 mL)** | | | | | | | | | | | |  |
| --- | --- | --- | --- | --- | --- | --- | --- | --- | --- | --- | --- | --- |
| A. Continuous outcomes | | | | | | | | | | | |  |
| Outcome | | | Time points | EPNS: LS-mean | | SNM: LS-mean | | Δ (EPNS–SNM), pp (95% CI) | | *P* | |  |
|  | | |  |  | |  | |  | |  | |  |
| PVR improvement rate (%) | | | T_1_ | 64 | | 47 | | 17 (3 to 31) | | 0.02 | |  |
|  |  |  | T_2_ | 81 | | 69 | | 12 (2 to 26) | | 0.1 | |  |
|  | | |  |  | |  | |  | |  | |  |
| ICIQ-LUTS improvement rate (%) | | | T_1_ | 26 | | 14 | | 12 (2 to 23) | | 0.03 | |  |
|  |  |  | T_2_ | 43 | | 15 | | 28 (17 to 39) | | <0.001 | |  |
|  | | |  |  | |  | |  | |  | |  |
| ICIQ-LUTSqol improvement rate (%) | | | T_1_ | 23 | | 9 | | 14 (7 to 20 ) | | <0.001 | |  |
|  |  |  | T_2_ | 36 | | 19 | | 17 (10 to 24) | | <0.001 | |  |
| B. Binary target outcomes | | | | | | | | | | | | |
|  | | | | | | | | | | | | |
| Target | Time points | EPNS: n/N，  risk % | | | SNM: n/N，  risk % | | RD, %  (Newcombe 95% CI) | | RR  (95% CI) | | *P* (Fisher) | |
|  |  |  | | |  | |  | |  | |  | |
| PVR<60mL | T_1_ | 11/35, 31 | | | 2/35, 6 | | 26 (0 to 46) | | 6 (1 to 23) | | 0.01 | |
|  | T_2_ | 16/35, 46 | | | 8/35, 23 | | 23 (-9 to 50) | | 2 (1 to 4) | | 0.08 | |
|  |  |  | | |  | |  | |  | |  | |
| PVR improvement≥50% | T_1_ | 20/35, 57 | | | 15/35, 43 | | 14 (-12 to 34) | | 1 (1 to 2) | | 0.3 | |
|  | T_2_ | 29/35, 83 | | | 24/35, 69 | | 14 (-14 to 40) | | 1 (1 to 2) | | 0.3 | |
|  |  |  | | |  | |  | |  | |  | |
| Composite | T_1_ | 11/35, 31 | | | 2/35, 6 | | 26 (0 to 46) | | 6 (1 to 23) | | 0.01 | |
|  | T_2_ | 16/35, 46 | | | 8/35, 23 | | 23 (-9 to 50) | | 2 (1 to 4) | | 0.08 | |
| Abbreviations: EPNS, electrical pudendal nerve stimulation; SNM, sacral neuromodulation; LS-mean, least-squares mean; RD, risk difference; RR, relative risk; CI, confidence interval; PVR, post-void residual; ICIQ-LUTS, International Consultation on Incontinence Questionnaire–Lower Urinary Tract Symptoms; ICIQ-LUTSqol, ICIQ-LUTS quality of life.  Assuming PVR ≤60 mL is physiologically normal, we subtracted 60 mL from each PVR measurement at all time points. Values ≤60 mL were recorded as 0, and values >60 mL were recorded as the measured value minus 60 mL. All PVR-based endpoints were recomputed under this definition. “Composite” denotes meeting both “PVR ≤60 mL” and “PVR improvement rate ≥50%” from baseline. | | | | | | | | | | | | |
| Panel A (continuous outcomes): Values are covariate-adjusted LS-means from linear mixed-effects models (random intercept; AR[1]); covariates: sex, age, symptom duration, lesion pattern, and baseline value of the corresponding outcome. Between-group contrasts at T_1_ and T_2_ are Bonferroni-adjusted within outcome. pp = percentage points. | | | | | | | | | | | | |
| Panel B (binary targets): Risks are shown as observed n/N and percentages. RD is reported with Newcombe 95% CIs; RR is reported with 95% CIs using the Haldane–Anscombe correction when applicable. P values are from two-sided Fisher’s exact tests. Denominators are observed cases; no imputation was used. | | | | | | | | | | | | |

| **Supplementary table 4– Panel A. Multivariable regression for primary and key secondary outcomes under the threshold-adjusted dataset (Threshold = 40 mL), in crude and propensity-matched cohorts** | | | | | | |
| --- | --- | --- | --- | --- | --- | --- |
| Outcome | Crude cohort — Model 1 β (95% CI) | *P* | Crude cohort — Model 2 β (95% CI) | *P* | Matched cohort — Model 1 β (95% CI) | *P* |
| PVR improvement rate | -0.16 (-0.23 to -0.09) | <0.001 | -0.13 (-0.26 to 0) | 0.06 | -0.12 (-0.27 to 0.04) | 0.2 |
|  |  |  |  |  |  |  |
| ICIQ-LUTS improvement rate | -0.29 (-0.4 to -0.18) | <0.001 | -0.28 (-0.40 to -0.17) | <0.001 | -0.30 (-0.43 to -0.17) | <0.001 |
|  |  |  |  |  |  |  |
| ICIQ-LUTSqol improvement rate | -0.16 (-0.23 to -0.09) | <0.001 | -0.17 (-0.25 to -0.1) | <0.001 | -0.17 (-0.26 to -0.08) | <0.001 |
| Assuming PVR ≤40 mL is physiologically normal, we redefined PVR at all time points by subtracting 40 mL from each measured value; values ≤40 mL were recorded as 0, and values >40 mL were recorded as the measured value minus 40 mL (e.g., 400 mL → 360 mL). All PVR-based endpoints were recomputed accordingly. ICIQ-LUTS and ICIQ-LUTSqol do not depend on the PVR threshold and are included for completeness and internal robustness | | | | | | |
| Abbreviations: CI, confidence interval; EPNS, electrical pudendal nerve stimulation; SNM, sacral neuromodulation; PVR, postvoid residual; ICIQ-LUTS, International Consultation on Incontinence Questionnaire–Lower Urinary Tract Symptoms; ICIQ-LUTSqol, International Consultation on Incontinence Questionnaire–Lower Urinary Tract Symptoms Quality of Life | | | | | | |
| β: β values are on the proportion scale for improvement-rate outcomes and represent the effect of SNM compared with EPNS (reference group); negative β indicates less improvement with SNM than with EPNS | | | | | | |
| Sample : crude cohort n=35; matched cohort n=24; the matched cohort was derived by propensity‑score matching | | | | | | |
| Model 1 adjusts for sex, age, duration, and lesion pattern. Model 2 additionally adjusts for baseline severity (PVR₀, ICIQ-LUTS₀, and ICIQ-LUTSqol₀) | | | | | | |

| **Supplementary table 4 – Panel B. Multivariable regression for primary and key secondary outcomes under the threshold-adjusted dataset (Threshold = 50 mL), in crude and propensity-matched cohorts** | | | | | | |
| --- | --- | --- | --- | --- | --- | --- |
| Outcome | Crude cohort — Model 1 β (95% CI) | *P* | Crude cohort — Model 2 β (95% CI) | *P* | Matched cohort — Model 1 β (95% CI) | *P* |
| PVR improvement rate | -0.12 (-0.24 to 0.01) | 0.07 | -0.12 (-0.25 to 0.00) | 0.06 | -0.14 (-0.30 to 0.02) | 0.09 |
|  |  |  |  |  |  |  |
| ICIQ-LUTS improvement rate | -0.29 (-0.40 to -0.18) | <0.001 | -0.28 (-0.40 to -0.17) | <0.001 | -0.32 (-0.45 to -0.200) | <0.001 |
|  |  |  |  |  |  |  |
| ICIQ-LUTSqol improvement rate | -0.16 (-0.23 to -0.09) | <.001 | -0.17 (-0.25 to -0.10) | <.001 | -0.20 (-0.29 to -0.12) | <0.001 |
| Assuming PVR ≤50 mL is physiologically normal, we subtracted 50 mL from each PVR measurement at all time points. Values ≤50 mL were recorded as 0, and values >50 mL were recorded as the measured value minus 50 mL. All PVR-based endpoints were recomputed under this definition Abbreviations, β interpretation, sample description, and model specifications are as in Panel A | | | | | | |

| **Supplementary table 4 – Panel C. Multivariable regression for primary and key secondary outcomes under the threshold-adjusted dataset (Threshold = 60 mL), in crude and propensity-matched cohorts** | | | | | | |
| --- | --- | --- | --- | --- | --- | --- |
| Outcome | Crude cohort — Model 1 β (95% CI) | *P* | Crude cohort — Model 2 β (95% CI) | *P* | Matched cohort — Model 1 β(95% CI) | *P* |
| PVR improvement rate | -0.11 (-0.24 to 0.02) | 0.111 | -0.12 (-0.25 to 0.00) | 0.06 | -0.14 (-0.30 to 0.02) | 0.1 |
|  |  |  |  |  |  |  |
| ICIQ-LUTS improvement rate | -0.29 (-0.40 to -0.18) | <0.001 | -0.28 (-0.40 to -0.17) | <0.001 | -0.32 (-0.45 to -0.20) | <0.001 |
|  |  |  |  |  |  |  |
| ICIQ-LUTSqol improvement rate | -0.16 (-0.23 to -0.09) | <0.001 | -0.17 (-0.25 to -0.10) | <0.001 | -0.20 (-0.29 to -0.12) | <0.001 |
| Assuming PVR ≤60 mL is physiologically normal, we subtracted 60 mL from each PVR measurement at all time points. Values ≤60 mL were recorded as 0, and values >60 mL were recorded as the measured value minus 60 mL. All PVR-based endpoints were recomputed under this definition  Abbreviations, β interpretation, sample description, and model specifications are as in Panel A | | | | | | |

| **Supplementary table 5 –Sensitivity analysis using untransformed absolute PVR volume as the outcome** | | | | | |
| --- | --- | --- | --- | --- | --- |
| Time points | EPNS: adjusted PVR, mL | SNM: adjusted PVR, mL | Difference, mL | 95% CI | *P* |
|  |  |  |  |  |  |
| T_1_ | 137 | 180 | -43 | -82 to -5 | 0.03 |
| T_2_ | 81 | 132 | -50 | -89 to -12 | 0.01 |
| Abbreviations: EPNS, electrical pudendal nerve stimulation; SNM, sacral neuromodulation; CI, confidence interval; PVR, post-void residual.  Difference = EPNS − SNM. Values are least-squares means from the adjusted linear mixed-effects model using untransformed absolute PVR volume at T_1_ and T_2_ as the dependent variable. The model included treatment group, time, and the group-by-time interaction, with adjustment for sex, age, symptom duration, lesion pattern, baseline PVR, baseline questionnaire outcomes scores. Pairwise between-group comparisons at each time point were Bonferroni-adjusted.  The group-by-time interaction was not significant (P=0.59). | | | | | |

**Supplementary methods 1: Standardized protocol for EPNS**

|  | **Prespecified protocol** | | | **Notes / allowed adjustment** |  |
| --- | --- | --- | --- | --- | --- |
| Treatment schedule | 12 sessions over 4 consecutive weeks | | | 3 sessions per week |  |
| Operator | Same licensed acupuncturist | | | To ensure procedural consistency |  |
| Pre-session preparation | Bladder emptying before each session | | | Performed before needle placement |  |
| Patient position | Prone | | | Fixed across sessions |  |
| Upper points | | Two points located approximately 1 cm lateral to the sacrococcygeal joint | Bilateral | |   TU |
| Lower points | | Two points located approximately 1 cm lateral to the coccygeal tip | Bilateral | |  |
| Upper-point needling | 0.40 × 100 mm needle, perpendicular insertion, depth 80–90 mm | | | Bilateral |   TU  |
| Lower-point needling | 0.40 × 125 mm needle, oblique insertion toward the ischiorectal fossa, depth 90–110 mm | | | Bilateral |  |
| Electrical pairing | Ipsilateral upper–lower needle pair connected as one circuit | | | Fixed configuration |  |
| Polarity | Upper needle = anode; lower needle = cathode | | | Fixed across sessions |  |
| Stimulator | Multipurpose electroacupuncture device (G6805-2, Shanghai, China) | | | Same device platform |  |
| Frequency | 2.5 Hz | | | Fixed |  |
| Current intensity | 25–35 mA | | | Titrated within prespecified range only |  |
| Intensity titration target | Tolerated urethral or anal referred sensation | | | No adjustment beyond prespecified range |  |
| Session duration | 60 min/session | | | Fixed |  |
| Adherence monitoring | Prospectively monitored throughout the intervention period | | |  |  |

**Supplementary methods 2: Standardized protocol for SNM**

|  | **Prespecified protocol** | **Notes / allowed adjustment** |
| --- | --- | --- |
| Treatment period | Continuous 4-week test phase | Matched to EPNS study duration |
| Target | S3 foramen | Standard target for test stimulation |
| Localization method | CT fusion imaging with a patient-specific 3-dimensional printed template | Used to assist S3 localization |
| Perioperative prophylaxis | Intravenous cefazolin 2g before the procedure | Standard prophylaxis |
| Anesthesia | Local anesthesia | Standardized |
| Needle insertion | Standard foramen needle inserted into the S3 foramen at approximately 60° | Procedural target fixed |
| Intraoperative stimulation testing | 14–35 Hz; pulse width 210–360 as | Used for confirmation of target placement |
| Confirmation of correct placement | Characteristic motor and sensory responses | Including bellows response and toe movement with concordant sensory feedback |
| Implanted lead | Quadripolar tined lead (Model 3889, Medtronic, Minneapolis, MN, USA) | Standard SNM lead |
| Test stimulator | External stimulator (InterStim II, Model 3058) | Used throughout test phase |
| Programming systems | InterStim ICON (Model 3037) and N’VISION (Model 8840) | Used for device programming and management |
| Postplacement verification | Radiographic and clinical verification of lead position | Before discharge |
| Postprocedure medication | Cephalexin 500 mg orally for 7 days | Standard postprocedure regimen |
| Monitoring during test phase | Monitoring for lead-site infection, lead-related complications, and other device-related adverse events | Throughout the 4-week period |
| Programming adjustment | Adjusted according to standard clinical practice to maintain effective and tolerable stimulation after confirmed lead placement | Allowed within routine test-phase management |
